# Association between cumulative duration of deep anesthesia and postoperative acute kidney injury after noncardiac surgeries: a retrospective observational study

**DOI:** 10.1080/0886022X.2023.2287130

**Published:** 2023-11-29

**Authors:** Wen-Kao Huang, Wan-Yi Lian, Xiao-Yu Zhuo, Song-Yun Kang, Wen-Chi Luo, Yi-Shan Xie, Gui-Yang Xi, Ke-Xuan Liu, Wei-Feng Liu

**Affiliations:** Department of Anesthesiology, Nanfang Hospital, Southern Medical University, Guangzhou, China

**Keywords:** Acute kidney injury, anesthetic depth, bispectral index, general anesthesia, noncardiac surgery

## Abstract

**Background:**

Bispectral index (BIS) is a processed electroencephalography monitoring tool and is widely used in anesthetic depth monitoring. Deep anesthesia exposure may be associated with multiple adverse outcomes. However, the relationship between anesthetic depth and postoperative acute kidney injury (AKI) remains unclear. We sought to determine the effect of BIS-based deep anesthesia duration on postoperative AKI following noncardiac surgery.

**Methods:**

This retrospective study used data from the Vital Signs DataBase, including patients undergoing noncardiac surgeries with BIS monitoring. The BIS values were collected every second during anesthesia. Restricted cubic splines and logistic regression were used to assess the association between the cumulative duration of deep anesthesia and postoperative AKI.

**Results:**

4774 patients were eligible, and 129 (2.7%) experienced postoperative AKI. Restricted cubic splines showed that a cumulative duration of BIS < 45 was nonlinearly associated with postoperative AKI (*P*-overall = 0.033 and *P*-non-linear = 0.023). Using the group with the duration of BIS < 45 less than 15 min as the reference, ORs of postoperative AKI were 2.59 (95% confidence interval [CI]:0.60 to 11.09, *p* = 0.200) in the 15–100 min group, and 4.04 (95%CI:0.92 to 17.76, *p* = 0.064) in the ≥ 100 min group after adjusting for preoperative and intraoperative covariates in multivariable logistic regression.

**Conclusions:**

The cumulative duration of BIS < 45 was independently and nonlinearly associated with the risk of postoperative AKI in patients undergoing noncardiac surgery.

## Introduction

Acute kidney injury (AKI) is a common and serious postoperative complication that may lead to prolonged hospital stay, increased medical costs, persistent renal failure, and in-hospital death [1–3]. A variety of preoperative risk factors contributing to the progression of postoperative AKI have been identified, such as American Society of Anesthesiologists (ASA) physical status ≥ III, anemia, comorbidities, and elevated risk surgery [4,5]. In addition, an increasing number of studies have indicated that intraoperative factors are closely related to the development of postoperative AKI, including risk factors such as intraoperative oliguria, perioperative nephrotoxic drug exposure, prolonged hypotension, and elevated central venous pressure [6–8]. Thus, optimizing perioperative management may reduce the risk of AKI and improve the postoperative outcomes.

The bispectral index (BIS) is derived from the analysis of electroencephalograms (EEGs), which in turn presents the anesthetic depth as a single value [9,10]. BIS monitors report numbers between 0 and 100, with 100 indicating an awake state and 0 indicating complete EEG inactivity. Studies have shown that cumulative duration of BIS below a certain threshold is associated with hospital stay and mortality [11,12]. General anesthesia involves different combinations and dosages of drugs that ensure unconsciousness, analgesia, and inhibition of potentially harmful hemodynamic responses [13]. Patients sensitive to anesthetics may receive higher dose of anesthetics than needed. Excessive drugs may aggravate the burden on the kidney and ultimately cause damage [14]. Previous studies have shown that intraoperative use of EEG and BIS data can better titrate the delivered anesthetics, thereby reducing the dosage of anesthetics and improving patient prognosis [15]. However, the relationship between anesthetic depth and postoperative acute kidney injury remains unclear. Identifying a novel association between intraoperative anesthetic depth and postoperative AKI may provide an opportunity to improve risk assessment and targeted interventions. Therefore, we conducted a retrospective observational study to explore the association between the cumulative duration of deep anesthesia and postoperative AKI following noncardiac surgery.

## Patients and methods

### Data source

We used the publicly available Vital Signs DataBase (VitalDB, https://vitaldb.net/) for our analysis, which included 6388 patients who underwent noncardiac surgical procedures at Seoul National University Hospital, Seoul, South Korea, from August 2016 to June 2017 [16]. Access to the database for research was approved by the Institutional Review Board of Seoul National University Hospital (H-1408-101-605). All patients in the database were anonymous, and the requirement for written informed consent was waived. The study was conducted in accordance with the principles set forth in the Helsinki Declaration, and the manuscript adhered to the STROBE statement guidelines.

### Ethics

Ethical approval for this study was not required because it used anonymized, publicly available data.

### Study population

All patients who underwent noncardiac surgical procedures were eligible for inclusion. Exclusion criteria were (i) patients with no baseline serum creatinine; (ii) patients did not receive general anesthesia; (iii) liver transplantation, kidney transplantation, nephrectomy, or surgeries for deceased patients (e.g., donor transplantation); (iv) preoperative estimated glomerular filtration rate (eGFR) < 15 mL/min/1.73 m^2^ based on the simplified MDRD formula; (v) no intraoperative BIS monitoring or record; and (vi) no intraoperative mean arterial pressure (MAP) record.

### Data collection

The following variables were selected a priori based on a literature review and extracted from the VitalDB database: age, sex, weight, ASA physical status classification, preoperative hypertension, preoperative diabetes mellitus, preoperative serum albumin, preoperative hemoglobin, preoperative serum creatinine, emergency operation, type of surgery, duration of anesthesia, intraoperative noninvasive and/or invasive MAP, intraoperative BIS value, intraoperative use of vasopressors, use of volatile anesthetic, intraoperative infusion and transfusion, length of stay (LOS), postoperative intensive care unit (ICU) admission, and in-hospital mortality. The following values were regarded as missing: (i) MAP was less than 20 mmHg or greater than 200 mmHg; (ii) BIS value of 0. The intraoperative BIS values were recorded every second. Noninvasive and invasive MAP data were acquired at 2 s interval. The MAP of the former second was used to fill a gap of 2 s. Invasive MAP values were primarily used for statistical analysis instead of noninvasive MAP values, if available. We defined deep anesthesia as a BIS value of less than 45 [11,12,17]. The cumulative duration of deep anesthesia and MAP below thresholds (55, 65, and 75 mmHg) were calculated for each patient.

### Outcome

The primary outcome was postoperative AKI (any stage), defined as an increase in serum creatinine by ≥ 0.3 mg/dl within 48 h after surgery or an increase to ≥ 1.5 baseline within 7 postoperative days [18]. Patients without creatinine measurements within seven days after the surgical procedure were assumed to have no occurrence of AKI.

### Statistical analyses

SPSS version 26.0 (IBM Corporation) and R (version 4.2.1; R Foundation for Statistical Computing) were used for the data analysis in this study. Patient characteristics are presented as median (interquartile range) or frequency (percentage), as appropriate. Non-normally distributed and categorical data were compared using the Kruskal-Wallis test and Pearson’s χ2 test (or Fisher’s exact test, if appropriate), respectively. Missing data was handled using multiple imputations.

To visualize the association of cumulative duration of BIS < 45 with postoperative AKI, we used unadjusted and adjusted restricted cubic spline (RCS) models with four knots (5th, 35th, 65th and 95th percentiles), with the cumulative duration = 0 as a reference. According to the shape of the association and 5th percentile, the cumulative duration of BIS < 45 was transformed into a categorical variable with three levels (< 15, 15-100 and ≥ 100 min). In the multivariable logistic regression analysis, all aforementioned variables, which have been checked for multicollinearity by calculating the variance inflation factor (< 10 for all), were included in the models. The criterion for statistical significance was set at *p* < 0.05 for all tests.

Several sensitivity analyses were performed to confirm the robustness of the results. Sensitivity models were constructed as adjusted RCS models mentioned above, including: (i) the cumulative duration of BIS < 40 was used as an independent variable to explore the relationship between the cumulative duration of deep anesthesia and postoperative AKI [12]; (ii) because the intraoperative hypotension threshold related to postoperative AKI was still uncertain, the model was adjusted for the duration of MAP below 75 mmHg; (iii) the model was adjusted for the duration of MAP below 55 mmHg; (iv) the model was adjusted for the duration of MAP 65-75 mmHg, 55-65 mmHg and < 55 mmHg; (v) patients without any postoperative serum creatinine measurements were excluded (*n* = 1397); (vi) patients with missing data were excluded (*n* = 264).

## Results

**Figure 1.**
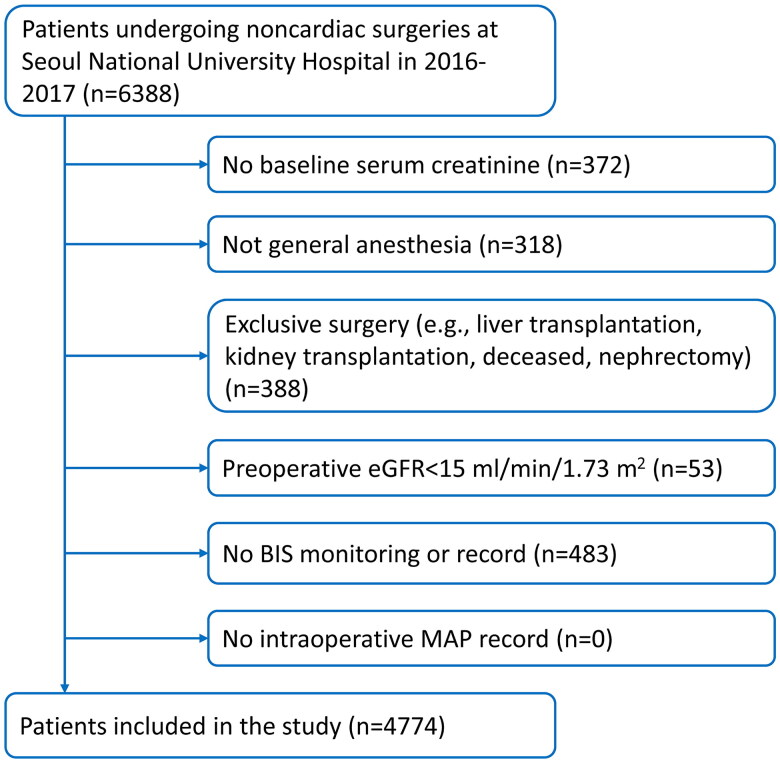
Study flow chart. BIS, bispectral index; eGFR, estimated glomerular filtration rate; MAP, mean arterial pressure.

Of the 6388 patients who underwent noncardiac surgeries identified in the VitalDB database, 4774 were eligible. The causes of exclusion are described in the flowchart (Figure 1). Among them, 129 (2.7%) experienced postoperative AKI. The characteristics of all included patients and grouping according to the duration of BIS below 45 are shown in Table 1. The results showed that except preoperative hemoglobin (*p* = 0.077) and in-hospital mortality (*p* = 0.328), all characteristics were significantly different among the three groups. Participants with longer duration of deep anesthesia had greater incidences of postoperative AKI (0.7% vs. 1.8% vs. 4.1%, respectively; *p* < 0.001) and ICU admission (*p* < 0.001), and had longer hospital LOS (*p* < 0.001).

**Table 1.** Patient characteristics according to duration of BIS below 45.

		Duration of BIS < 45 (min)				
	Overall	< 15	≥ 15 and < 100	≥ 100	*P*-value	Missing
Characteristic**s**	*n* = 4774	*n* = 291	*n* = 2443	*n* = 2040		data (%)
Age (y)					**< 0.001**	0
< 40	496 (10.4)	43 (14.8)	288 (11.8)	165 (8.1)		
≥ 40 and < 65	2555 (53.5)	159 (54.6)	1348 (55.2)	1048 (51.4)		
≥ 65	1723 (36.1)	89 (30.6)	807 (33.0)	827 (40.5)		
Gender (male)	2340 (49.0)	113 (38.8)	1053 (43.1)	1174 (57.5)	**< 0.001**	0
Weight (kg)	60.5 (53.4–68.5)	58.9 (52.6–66.1)	60.0 (53.4–68.5)	61.3 (53.7–68.9)	**0.023**	0
ASA physical status					**0.012**	0
I-II	4332 (90.7)	269 (92.4)	2241 (91.7)	1822 (89.3)		
III-IV	442 (9.3)	22 (7.6)	202 (8.3)	218 (10.7)		
Hypertension	1475 (30.9)	77 (26.5)	717 (29.3)	681 (33.4)	**0.003**	0
Diabetes mellitus	522 (10.9)	18 (6.2)	228 (9.3)	276 (13.5)	**< 0.001**	0
Preoperative serum albumin (g/l)	42 (39–44)	41 (38–44)	42 (39–44)	41 (38–44)	**< 0.001**	< 0.1
Preoperative hemoglobin (g/l)	131 (118–142)	128 (116–140)	131 (118–141)	131 (118–143)	0.077	0.2
Preoperative eGFR (ml/min/1.73 m^2^)					**0.009**	0
≥ 90	2821 (59.1)	199 (68.4)	1452 (59.4)	1170 (57.4)		
≥ 60 and < 90	1710 (35.8)	75 (25.8)	870 (35.6)	765 (37.5)		
≥ 30 and < 60	213 (4.5)	14 (4.8)	109 (4.5)	90 (4.4)		
≥ 15 and < 30	30 (0.6)	3 (1.0)	12 (0.5)	15 (0.7)		
Emergency	475 (9.9)	35 (12.0)	270 (11.1)	170 (8.3)	**0.005**	0
Type of surgery					**< 0.001**	0
General	3527 (73.9)	234 (80.4)	1880 (77.0)	1413 (69.3)		
Thoracic	826 (17.3)	39 (13.4)	376 (15.4)	411 (20.1)		
Gynecologic	191 (4.0)	6 (2.1)	108 (4.4)	77 (3.8)		
Vascular	141 (3.0)	9 (3.1)	54 (2.2)	78 (3.8)		
Urologic	89 (1.9)	3 (1.0)	25 (1.0)	61 (3.0)		
Duration of anesthesia (h)					**< 0.001**	0
< 2	1214 (25.4)	166 (57.0)	1048 (42.9)	0 (0)		
≥ 2 and ≤ 5	2828 (59.2)	106 (36.4)	1331 (54.5)	1391 (68.2)		
> 5	732 (15.3)	19 (6.5)	64 (2.6)	649 (31.8)		
Duration of MAP < 75 mmHg (min)	35.7 (16.0–68.6)	29.2 (14.8–51.9)	27.3 (12.0–51.4)	50.4 (24.8–92.4)	**< 0.001**	0
Duration of MAP < 65 mmHg (min)	7.6 (1.8–19.3)	8.6 (2.0–22.4)	6.0 (0.9–15.3)	9.8 (2.5–23.2)	**< 0.001**	0
Duration of MAP < 55 mmHg (min)	0.4 (0–2.2)	0.4 (0–3.2)	0.1 (0–1.6)	0.9 (0.1–2.5)	**< 0.001**	0
Intraoperative vasopressors	2725 (57.1)	170 (58.4)	1216 (49.8)	1339 (65.6)	**< 0.001**	0
Volatile anesthetic	2519 (52.8)	148 (50.9)	1153 (47.2)	1218 (59.7)	**< 0.001**	0
Intraoperative crystalloid (ml)	700 (400–1300)	400 (210–700)	450 (300–787)	1200 (750–1850)	**< 0.001**	5.2
Intraoperative colloid	272 (5.7)	8 (2.7)	65 (2.7)	199 (9.8)	**< 0.001**	0
Intraoperative RBC transfusion	218 (4.6)	7 (2.4)	54 (2.2)	157 (7.7)	**< 0.001**	0
Intraoperative FFP transfusion	57 (1.2)	2 (0.7)	8 (0.3)	47 (2.3)	**< 0.001**	0
Postoperative AKI	129 (2.7)	2 (0.7)	44 (1.8)	83 (4.1)	**< 0.001**	0
LOS (d)	7 (4–10)	5 (3–8)	6 (3–8)	9 (7–13)	**< 0.001**	0
Postoperative ICU admission	852 (17.8)	25 (8.6)	247 (10.1)	580 (28.4)	**< 0.001**	0
In-hospital mortality	42 (0.9)	2 (0.7)	17 (0.7)	23 (1.1)	0.328	0

Data are median (inter-quartile range) or *n* (%). AKI: acute kidney injury; ASA: American society of anesthesiologists; BIS: bispectral index; eGFR: estimating glomerular filtration rate; FFP: fresh frozen plasma; ICU: intensive care unit; LOS: length of stay; MAP: mean arterial pressure; RBC: red blood cell.

**Figure 2.**
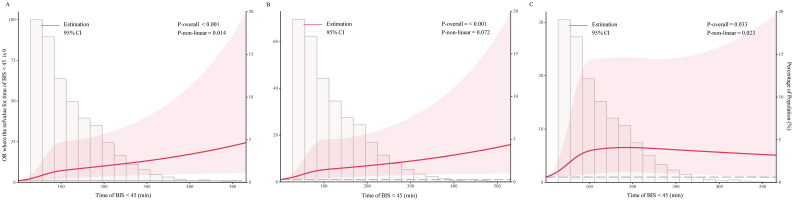
Association between the cumulative duration of BIS < 45 and postoperative AKI. Restricted cubic spline models were used to visualize the shape, with 0 min as the reference value. Solid lines and shaded areas denote ORs and 95% CIs, respectively. Figure 2A, unadjusted. Figure 2B, adjusting for preoperative covariates. Figure 2C, adjusting for preoperative and intraoperative covariates. AKI, acute kidney injury; BIS, bispectral index; CI, confidence interval; OR, odds ratio; refvalue, reference value.

Restricted cubic splines were used to visualize the relationship between the cumulative duration of BIS < 45 and the risk of postoperative AKI (Figure 2). A clear dose–response relationship was observed between the duration of deep anesthesia and AKI in the univariate model (Figure 2A). The risk of AKI increased with the amount of time spent below the BIS threshold (*P*-overall < 0.001, *P*-non-linear = 0.014). Adjusting for potentially confounding preoperative covariates did not appreciably change the relationship between the two variables (*P*-overall < 0.001, *P*-non-linear = 0.072, Figure 2B). After adjusting for preoperative and intraoperative covariates (including the duration of MAP below 65 mmHg), a significant nonlinear correlation between the two variables was observed (*P*-overall = 0.033, *P*-non-linear = 0.023, Figure 2C). When the duration of BIS below the threshold was less than 100 min, the results showed a positive correlation between them, while the curve presented a plateau after 100 min.

Considering the nonlinear associations, the duration of BIS < 45 was converted into a three-level categorical variable (< 15, 15-100 and ≥ 100 min) in the multivariable logistic regression analysis (Table 2). Using the group with the duration of BIS below threshold < 15 min as the reference, the ORs of postoperative AKI were 2.50 (95%CI:0.59 to 10.55, *p* = 0.212) in the 15-100 min group, and 5.08 (95%CI:1.22 to 21.18, *p* = 0.026) in the ≥ 100 min group after adjusting for preoperative covariates in model 1. In model 2, the ORs were 2.59 (95%CI:0.60 to 11.09, *p* = 0.200) in the 15-100 min group, and 4.04 (95%CI:0.92 to 17.76, *p* = 0.064) in the ≥ 100 min group after adjusting for preoperative and intraoperative covariates.

**Table 2.** Association between cumulative duration of BIS below 45 and postoperative AKI.

Variables in the model	Model 1		Model 2	
	aOR (95%CI)	*p*-value	aOR (95%CI)	*p*-value
Age (y)				
< 40	1 (reference)		1 (reference)	
≥ 40 and < 65	0.93 (0.45 to 1.94)	0.850	1.01 (0.47 to 2.15)	0.982
≥ 65	0.68 (0.31 to 1.49)	0.340	0.70 (0.32 to 1.57)	0.392
Gender (male)	3.11 (1.96 to 4.94)	**< 0.001**	2.81 (1.74 to 4.53)	**< 0.001**
Weight (kg)	0.99 (0.97 to 1.01)	0.372	0.99 (0.97 to 1.01)	0.266
ASA physical status				
I-II	1 (reference)		1 (reference)	
III-IV	2.14 (1.37 to 3.34)	**< 0.001**	2.17 (1.36 to 3.46)	**0.001**
Hypertension	1.66 (1.10 to 2.49)	**0.015**	1.65 (1.09 to 2.50)	**0.019**
Diabetes mellitus	1.36 (0.85 to 2.18)	0.206	1.33 (0.82 to 2.15)	0.248
Preoperative serum albumin (g/l)	0.48 (0.33 to 0.71)	**< 0.001**	0.54 (0.36 to 0.80)	**0.003**
Preoperative hemoglobin (g/l)	0.83 (0.74 to 0.93)	**0.001**	0.82 (0.72 to 0.92)	**< 0.001**
Preoperative eGFR (ml/min/1.73 m^2^)				
≥ 90	1 (reference)		1 (reference)	
≥ 60 and < 90	0.72 (0.45 to 1.14)	0.165	0.69 (0.43 to 1.11)	0.129
≥ 30 and < 60	1.20 (0.61 to 2.35)	0.597	1.17 (0.58 to 2.35)	0.666
≥ 15 and < 30	1.38 (0.46 to 4.14)	0.561	1.52 (0.50 to 4.64)	0.464
Emergency	1.21 (0.73 to 1.99)	0.464	1.23 (0.72 to 2.10)	0.447
Type of surgery				
General			1 (reference)	
Thoracic			0.93 (0.55 to 1.59)	0.799
Gynecologic			0.53 (0.07 to 4.00)	0.536
Vascular			1.00 (0.43 to 2.36)	0.991
Urologic			5.29 (2.19 to 12.76)	**< 0.001**
Duration of anesthesia (h)				
< 2			1 (reference)	
≥ 2 and ≤ 5			0.94 (0.49 to 1.78)	0.846
> 5			1.13 (0.49 to 2.60)	0.768
Duration of MAP < 65 mmHg (min)			1.01 (1.00 to 1.01)	**0.014**
Intraoperative vasopressors			0.82 (0.53 to 1.28)	0.381
Duration of BIS < 45 (min)				
< 15	1 (reference)		1 (reference)	
≥ 15 and < 100	2.50 (0.59 to 10.55)	0.212	2.59 (0.60 to 11.09)	0.200
≥ 100	5.08 (1.22 to 21.18)	**0.026**	4.04 (0.92 to 17.76)	0.064
Volatile anesthetic			0.94 (0.63 to 1.41)	0.777
Intraoperative crystalloid (ml)			1.00 (1.00 to 1.00)	**0.038**
Intraoperative colloid			1.12 (0.62 to 2.02)	0.702
Intraoperative RBC transfusion			1.24 (0.65 to 2.38)	0.520
Intraoperative FFP transfusion	:		0.67 (0.25 to 1.83)	0.438

AKI: acute kidney injury; aOR: adjusted odds ratio; ASA: American Society of Anesthesiologists; BIS, bispectral index; CI, confidence interval; eGFR: estimating glomerular filtration rate; FFP: fresh frozen plasma; MAP: mean arterial pressure; RBC: red blood cell.

In Model 1, multivariable logistic regression adjusted for preoperative variables and the cumulative duration of BIS below 45. In Model 2, multivariable logistic regression adjusted for preoperative and intraoperative variables.

**Figure 3.**
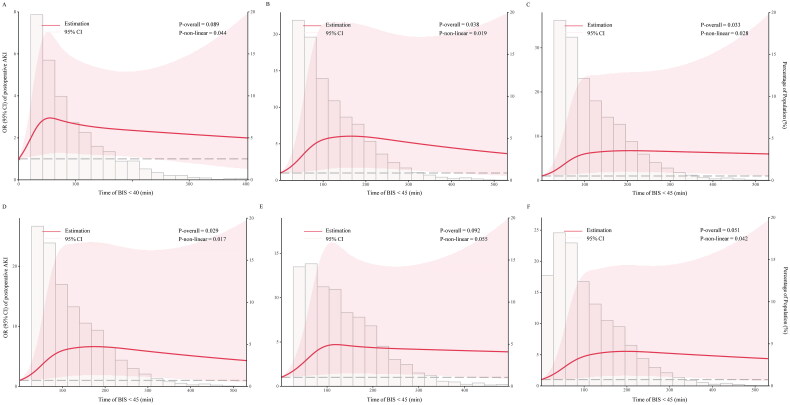
Sensitivity analysis. Solid lines and shaded areas denote ORs and 95% CIs, respectively. The reference value was 0 min. Figure 3A, restricted cubic splines for postoperative AKI according to the cumulative duration of BIS < 40. Figure 3B, adjusted for preoperative variables, the duration of MAP below 75 mmHg and other intraoperative variables. Figure 3C, adjusted for preoperative variables, the duration of MAP below 55 mmHg and other intraoperative variables. Figure 3D, adjusted for preoperative variables, the duration of MAP 65-75 mmHg, 55-65 mmHg and < 55 mmHg, and other intraoperative variables. Figure 3E, excluding patients without any postoperative Cr measurements. Figure 3F, excluding patients with missing data. AKI, acute kidney injury; BIS, bispectral index; CI, confidence interval; OR, odds ratio.

The results of the sensitivity analysis are shown in (Figure 3). First, after redefining deep anesthesia as BIS less than 40, the ORs of AKI increased with the duration of BIS below the threshold when the duration was less than 50 min. Subsequently, the curve plateaued (*P*-overall = 0.089, *P*-non-linear = 0.044, Figure 3A). Second, after adjustment for the duration of MAP below 75 mmHg, a significant association between the duration of BIS < 45 and the outcome was replicated (*P*-overall = 0.038, *P*-non-linear = 0.019, Figure 3B). Third, after adjusting for the duration of MAP below 55 mmHg, the association remained significant (*P*-overall = 0.033, *P*-non-linear = 0.028, Figure 3C). Fourth, after simultaneously adjusting for the duration of MAP < 55, 55-65 and 65-75 mmHg, the correlation was still significant (*P*-overall = 0.029, *P*-non-linear = 0.017, Figure 3D). Fifth, after removing patients without any postoperative serum creatinine measurements, the shape of the dose-response curve was not significantly modified (*P*-overall = 0.092, *P*-non-linear = 0.055, Figure 3E). Sixth, when patients with missing data were excluded, the shape of the curve did not change significantly (*P*-overall = 0.051, *P*-non-linear = 0.042, Figure 3F)

## Discussion

We report a retrospective observational study that evaluated the influence of the cumulative duration of deep anesthesia on postoperative AKI. Our results suggest that the cumulative duration of BIS < 45 was independently nonlinearly associated with postoperative AKI in patients undergoing noncardiac surgeries. This finding has important implications in clinical practice because anesthetic depth is one of the few modifiable intraoperative risk factors for adverse outcomes.

Previous reports have found strong correlations between deep anesthesia exposure and mortality [11,12,19]. Kertai et al. found an association between the cumulative duration of low BIS and mortality after cardiac surgery, and in addition, Sessler et al. confirmed that low BIS was a strong predictor for mortality when combined with low MAP and low minimum alveolar concentration fraction [11,12]. The vast majority of studies including the former two tended to lock their gaze to the mortality. Some important outcomes including postoperative AKI are often viewed as “intermediate outcomes” and do not attract enough attention from clinicians and researchers. No study has focused on the relationship between deep anesthesia and postoperative AKI, which is strongly associated with mortality [20]. Therefore, in our study, we turned our attention to postoperative AKI, which might serve as a mediating factor in the effect of low BIS on death. We found that the risk of postoperative AKI appeared to be associated with the cumulative duration of deep anesthesia (BIS less than 45) in a dose-dependent manner in the range of 0-100 min, whereas beyond this range, the risk did not further increase. Therefore, anesthetic depth deserves increased attention from anesthesiologists as a significant prognostic factor.

There are several potential causes of low intraoperative BIS. First, a low BIS value could be a direct consequence of an excessive anesthetic dose. Second, at equivalent doses of anesthetics, patients who are sensitive to anesthetics are more likely to be exposed to deep anesthesia. Third, inadequate cerebral perfusion could be a potential cause of low BIS. Therefore, we focused on the duration of intraoperative hypotension in our analysis because this variable may have direct effects on both low BIS and the primary outcome. We used multivariable analyses adjusted for the intraoperative cumulative duration of hypotension with various definitions to assess the relationship between low BIS and postoperative AKI. The results revealed that the effect of a low BIS on postoperative AKI may be independent of systemic hypotension. Nevertheless, it should still be viewed with cautious, for the reason that we cannot completely rule out the possibility of residual confounding from unmeasured factors (e.g., postoperative hypotension).

The incidence of postoperative AKI observed in this study (2.7%) was lower than that reported in the literature (2.9–57.4%) [7]. The differences could be partially explained by remaining patients without postoperative creatinine measurements, who were considered as a low-risk population, and excluding those undergoing high-risk surgical procedures (e.g., liver transplantation). In our analysis, we found that the cumulative duration of low BIS was significantly related to the risk of postoperative AKI. There are several possible explanations for this finding. First, higher anesthetic vulnerability (i.e., lower BIS at a similar anesthetic dose) could be a manifestation of frailty, and the latter is considered an important risk factor for AKI [21,22]. Second, low BIS could be a direct consequence of excessive anesthetic administration, which aggravates the metabolic burden of the kidney and, in turn, increases the risk of adverse outcomes, including postoperative AKI.

Our study had some limitations. Considering the diversity and complexity of anesthetic regimens, the intraoperative anesthetic dose was not included in our analysis. Therefore, it remains unclear whether the association between the cumulative duration of low BIS and postoperative AKI is independent of anesthetic dose. In addition, our conclusions may be limited by the retrospective design of the study. We cannot fully rule out the possibility that residual confounding affects our results, even though we performed a number of sensitivity analyses. Furthermore, valid subgroup analyses could not be adequately carried out in our study due to the limited sample size in some subgroups.

In summary, we found that a cumulative duration of BIS < 45 was independently associated with an increased risk of postoperative AKI in patients undergoing non-cardiac surgery. Optimizing anesthetic depth may represent a modifiable and effective strategy to prevent postoperative AKI.

## Data Availability

The data underlying this article are available in the publicly available Vital Signs Database (https://vitaldb.net/).
